# Inhibitory Effect of *Lactobacillus plantarum* CCFM8724 towards *Streptococcus mutans*- and *Candida albicans*-Induced Caries in Rats

**DOI:** 10.1155/2020/4345804

**Published:** 2020-12-19

**Authors:** Qiuxiang Zhang, Sujia Qin, Xianyin Xu, Jianxin Zhao, Hao Zhang, Zhenmin Liu, Wei Chen

**Affiliations:** ^1^State Key Laboratory of Food Science and Technology, Jiangnan University, Wuxi, Jiangsu 214122, China; ^2^State Key Laboratory of Dairy Biotechnology, Shanghai Engineering Research Center of Dairy Biotechnology, Dairy Research Institute, Bright Dairy & Food Co., Ltd., Shanghai 200436, China; ^3^School of Food Science and Technology, Jiangnan University, Wuxi, Jiangsu 214122, China; ^4^Department of Stomatology, Wuxi Children's Hospital, Wuxi, Jiangsu 214023, China; ^5^National Engineering Research Center for Functional Food, Jiangnan University, Wuxi, Jiangsu 214122, China; ^6^Wuxi Translational Medicine Research Center and Jiangsu Translational Medicine Research Institute Wuxi Branch, China; ^7^Beijing Innovation Centre of Food Nutrition and Human Health, Beijing Technology and Business University (BTBU), Beijing 100048, China

## Abstract

*Streptococcus mutans* is a recognized cariogenic bacterium and a major producer of biofilm matrix. The presence of *Candida albicans* in dental plaque with *S*. *mutans* enhances the virulence leading to the onset of rampant caries which is similar to early childhood caries (ECC). The purpose of this study was to explore the effect of *Lactobacillus plantarum* CCFM8724 (CCFM8724) on the treatment and prevention of dental caries induced by *S. mutans* and *C. albicans in vivo*. Rats were divided into 6 groups: the control group and model group, 2 treatment groups, and 2 prevention groups (0.02% chlorhexidine or CCFM8724). The fluctuation of microbial colonization and the change of bacteria flora in rat oral cavity after sowing of *L*. *plantarum* CCFM8724 were investigated by colony-forming units (CFU) and microflora analysis. The caries of rats were assessed by microcomputed tomography (micro-CT) and Keyes scoring method. The results showed that *L. plantarum* CCFM8724 in both the treatment and prevention groups could significantly decrease the population of *S. mutans* and *C. albicans* in the rats' oral cavity (*p* < 0.001), the mineral loss of enamel (*p* < 0.05), and the scores of caries (*p* < 0.05). Besides, *L*. *plantarum* CCFM8724 exhibited better effects than chlorhexidine. Hence, *L. plantarum* CCFM8724 was proved to be a potential oral probiotic on caries treatment and prevention *in vivo* and it may have the prospect of application in dental caries (especially ECC) prevention products.

## 1. Introduction

Dental caries is one of the most prevalent oral bacterial infectious diseases that afflict children and adults worldwide. It can lead to the irreversible tooth demineralization, which is mainly associated with acidic biofilm formation and sugar intake [1]. Early childhood caries (ECC) is a unique form of rampant caries developed in babies and toddlers, especially preschool children. ECC can result in rapid and aggressive destruction of primary teeth which not only causes painful pulpal but also impacts on the development of permanent dentition and systemic health [2, 3]. Traditional treatment for deciduous caries is extremely costly and time consuming. Hence, ECC has been a major challenge in public health until today.

*Streptococcus mutans* has been recognized as the main cariogenic bacteria for its strong acidogenic and aciduric capacities [4]. And it is also the major producer of biofilm matrix, providing acid milieus within which caries-associated organisms thrive [5]. Intriguingly, recent studies have found that, in addition to *S. mutans*, fungus such as *Candida albicans* was detected frequently in high numbers in plaque biofilms from toddlers with caries [6, 7]. Moreover, the presence of *C. albicans* in mixed-species biofilms induces *S. mutans* glucosyltransferase (GTF) expression, which converts dietary sucrose into extracellular polysaccharides (EPS) and build more cariogenic biofilms [8]. Rather, the synergistic alliance of them amplifies the biofilm virulence which led to rampant caries similar to ECC in an animal model [9].

Common mechanical measures for dental caries control, although effective, have been limited by the preference of children and the elderly. It is known that chlorhexidine (CHX) has a bactericidal effect on *S. mutans* and fungicidal effect on *C. albicans* [10]. While the limitation of antimicrobial drugs is that once the intervention with CHX stops, the same pathogens will repopulate in oral niches and result in recurrence of tooth decay. To avoid this, lower dental plaque virulence with ecological methods are being increasingly preferred over broad-spectrum antimicrobials for long-term caries control [11]. In this regard, it is essential to develop an alternative product which can disrupt the cariogenic virulence and promote oral microecological balance with no adverse effects.

The FAO/WHO (Food and Agriculture Organization of the United Nations, World Health Organization) define probiotics as live microorganisms that play a beneficial role in the health of the host by taking appropriate amounts. In the past 10 years, probiotics have achieved a lot in preventing or treating gastrointestinal infections and diseases such as acute diarrhea, ulcerative colitis, Crohn's disease, and pouchitis [12, 13]. Currently, with the increasing acceptance of probiotics, more and more attention has been paid to probiotic usage in other areas such as dental caries prevention, periodontitis relieving, and regulation of oral microecological balance [14]. And several clinical trials have demonstrated that the administration of *Lactobacillus* genus including *L. rhamnosus*, *L. reuteri*, and *L. paracasei* decreased the lesion of caries in children [15–17]. However, the number of studies on the interaction of *Lactobacillus* with cariogenic bacteria and fungi and the effects of the probiotic *L. plantarum* on ECC caries model *in vivo* is limited, which prompted us to address these knowledge gaps.

In our previous study, *L. plantarum* CCFM8724 isolated from healthy human feces exhibited a considerable effect on inhibiting *S. mutans* and *C. albicans* dual biofilm formation *in vitro* [18]. In this study, we examined the changes of *S. mutans* and *C. albicans* colonization and caries development in the rat oral cavity by plate culture counting, 16S rDNA sequencing, micro-CT, and caries scoring to evaluate the effect of *L. plantarum* CCFM8724 on the prevention or treatment of caries *in vivo*. These results will provide enlightenment and proof on probiotics preventing and treating rampant caries induced by *S. mutans* and *C. albicans*.

## 2. Methods

### 2.1. Ethics Statement

This research methodology was conducted in accordance with institutional ethical standards. The study on animals was approved by “Jiangsu Institute of Parasitic Diseases, Animal Care and Use Committee” (IACUC-JIPD-2019030). All experiments were in accordance with the guidelines of the China Ministry of Science and Technology Guide for the Care and Use of Laboratory Animals.

### 2.2. Strains and Inoculum Preparation

*L*. *plantarum* CCFM8724 was isolated from healthy human feces and inoculated in MRS broth (Difco™, Detroit, MI, USA) under anaerobic conditions at 37°C. *S. mutans* ATCC 25175 was purchased from the China Common Microbial Species Preservation and Management Center (CGMCC, Beijing, China) and cultured in Tryptic Soy Broth (TSB, Difco™, Detroit, MI, USA) under anaerobic conditions at 37°C. *C. albicans* SJ was isolated from human carious dentin, which was grown in Yeast Extract Peptone Dextrose Medium (YPD, Difco™, Detroit, MI, USA) under aerobic conditions at 37°C. All strains were frozen in 30% (*v*/*v*) glycerol broth at -80°C and routinely streaked on corresponding agar plates. The plates were cultured at 37°C for 48 h and at least three times consecutively using 2% (*v*/*v*) inoculum in corresponding broth at 37°C for 18 h before use.

Prior to use in animal experiments, the microbial cultures were centrifuged at 3000 g for 10 min and washed twice with sterile saline solution. The bacteria and yeast were then centrifuged and resuspended in saline solution and diluted to a suspension of 1 × 10^9^ living cells (CCFM8724 and *S*. *mutans*) or 10^6^ (*C. albicans*) by colony counting.

### 2.3. Animals and General Procedures

**Figure 1:**
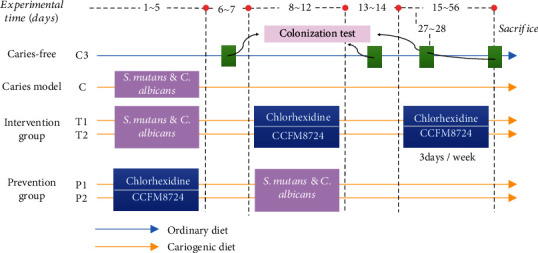
Animal experimental design.

Animal experimental design is shown in Figure 1. Female SPF Wistar rats (21 days old) were purchased from Charles River Laboratories (Beijing, China). All of the rats were divided randomly into 6 groups, comprising of 2 treatment groups (T1-CHX and T2-CCFM8724), 2 prevention groups (P1- CHX and P2-CCFM8724), and the caries-free and caries model groups. The rats in the caries-free group were given normal diet and distilled water during the whole experiment. Other groups were offered a cariogenic diet 2000 (obtained from Nantong Trophy Feed Technology Co., Ltd.) and water containing 5% sucrose *ad libitum*. The oral flora of rats was suppressed by ampicillin (0.5 *μ*g/mL) and streptomycin (200 *μ*g/mL) for 3 days before the experiment.

The caries model group was infected with *S. mutans* and *C. albicans* for 5 consecutive days from the first day of the experiment. The specific manipulation was to saturate the sterile cotton sticks with suspension of the two cariogenic microorganisms separately and then coat the suspension onto the rat oral cavity for 15 s per quadrant, as described by Beiraghi et al. [19]. Diet and water were forbidden for half an hour after tooth coating to ensure the colonization of microorganisms [20]. The establishment of tested strains was verified on day 6 and 7. The treatment groups were then applied with 0.02% CHX or CCFM8724 from day 8 to 12 and day 15 to 56. After verifying the successful colonization of lactobacilli, CHX or *Lactobacillus* was given three times a week until the end of the experiment (from day 15 to 56).

To test the prevention effect of *Lactobacillus* on caries, CHX or CCFM8724 was firstly applied to coat the teeth on day 1-5. After the establishment of lactobacilli, *S. mutans* and *C. albicans* were used for another 5 consecutive days. No treatment was done in the prevention groups for the next 6 weeks to the end of the experiment. The whole experiment period was 8 weeks. The rats were weighed daily, and their weight gains were calculated.

### 2.4. Oral Microbial Count

Four samples (day 6, 13, 27, and 56) were taken during the whole experiment. Specific sampling method was scraping the surface of rat dentin with sterile cotton stick back and forth three to four times. *S. mutans* was counted on Mitis Salivarius Agar (Difco, No. 229810, BD Diagnostic Systems) with 200 IU/L bacitracin (MSB) [21], and *C. albicans* was plated on BIGGY Agar (BBL, No. 211027, BD Diagnostic Systems) [22], while MRS supplemented with 12 *μ*g/mL vancomycin was used to enumerate lactobacilli [23].

### 2.5. DNA Extraction, PCR, and 16S rDNA Sequencing

Cotton swabs sampled from the rat oral cavity on day 56 before sacrificed were stored at -80°C before examination. According to the instructions, FastDNA Spin Kit for feces (MP Biomedical, United States) was used to extract microbial genome DNA. The V3-V4 region of the 16S rDNA gene was amplified by PCR. After cutting from the 1.5% agarose gel, the product was purified by a QIAquick Gel Extraction Kit (Qiagen, Germany) and quantified by a Quant-iT PicoGreen dsDNA Assay Kit (Life Technologies, United States). Libraries were established by a TruSeq DNA LT Sample Preparation Kit (Illumina, United States) and were sequenced for 500 + 7 cycles on Illumina MiSeq using a MiSeq Reagent Kit. At last, the sequence data of 16S rDNA were analyzed by QIIME pipeline as described previously [24].

### 2.6. Caries Scoring

On day 56, the rats were anesthetized and decapitated. The soft tissues on the teeth and jaws were peeled off with a scalpel, and the residual debris in the sutures was washed by ultrasonic cleaning for 20 minutes. The maxillary and mandibular molars were soaked in 10% polyformaldehyde for 24 hours [25, 26], then washed and dried. All the specimens were immersed in a 0.4% ammonium purpurate staining solution for 12 h and hemisectioned in the mesiodistal direction with an ultrathin carborundum disk (0.2 mm in thickness). The caries of rat molars were observed under a stereomicroscope (Leica CLS 100X, Wetzlar, Germany) and scored according to the method reported by Keyes [27].

### 2.7. Micro-CT Analysis

All mandibles were imaged using a microcomputed tomography (micro-CT) system (Quantum GX2; PerkinElmer, Hopkinton, MA, USA). An acquisition setting was used for scanning all the samples: 90 KV, 88 *μ*A; field of view: 18 *μ*m; acquisition time: 4 min; camera mode: high resolution. Each sample was rotated 360°, and all images were imported into Analyze 12.0 software (AnalyzeDirect, Overland Park, KS, USA) to reconstruct three-dimensional images of the mandibles, respectively. And a fixed threshold of 5,200 Hounsfield units was used to separate enamel from the whole mandible [28]. Mineral density (MD) of the enamel was calculated after calibration with hydroxyapatite standards of appropriate density.

### 2.8. Statistical Analysis

SPSS Statistics 25.0 (SPSS, Inc., Chicago, IL, USA) was used for the analysis. Graphpad Prism 8.0 and Origin 8.5 were used to map and analyze the data. The differences between the mean values of the groups were analyzed by one-way analysis of variance using Duncan's multiple range tests. Data expressed as mean ± standard error of the mean (s.d, *n* = 8).

## 3. Results

### 3.1. Microorganism Colonization of the Rat Oral Cavity

**Figure 2:**
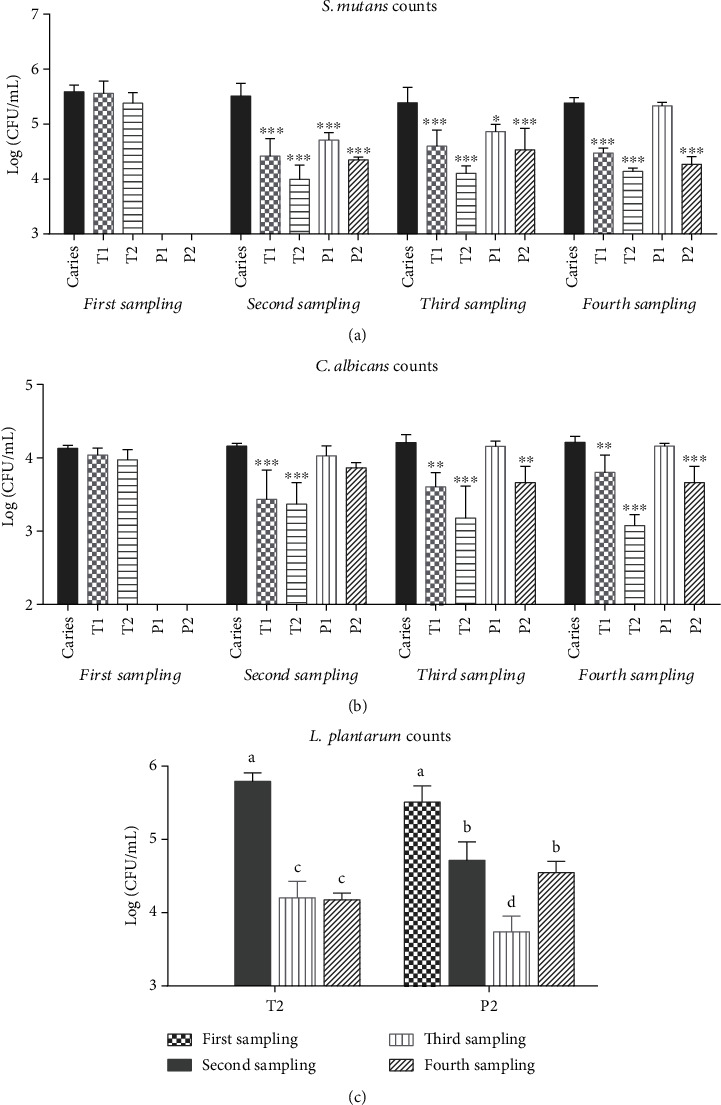
Microorganism count (log CFU/mL) recovered from rat oral swabs for 4 times. (a) Counting results of S. *mutans.* (b) Counting results of *C*. *albicans.* Values are significantly different from the caries model group in each sampling at ^∗^*p* < 0.05, ^∗∗^*p* < 0.01, or ^∗∗∗^*p* < 0.001 (a, b). (c) Counting results of *L*. *plantarum.* Groups with dissimilar letters differ, *p* < 0.05.

During the animal experiment, all animals appeared to be in good physical condition and no significant differences in weight gain were observed among the groups (*p* > 0.05). The colonization of *S. mutans* and *C. albicans* were 5.6 and 4.1 log (CFU/mL) in the first sampling, respectively (Figures 2(a) and 2(b)). After 5 consecutive days of *Lactobacillus* intervention, the population of *S. mutans* in T2 groups decreased from 5.4 to 4.0 log (CFU/mL) (*p* < 0.001) and remained at 4.1 log (CFU/mL) until the end of the experiment. CHX (T1) also inhibited the growth of *S. mutans* throughout the experiment, which reduced the population of *S. mutans* to 4.5 log (CFU/mL) (*p* < 0.001) and stabilized at this level.

After administered 0.02% CHX or CCFM8724 on day 1-5, the colonization of *S. mutans* in the prevention groups (P1 and P2) was significantly lower than that in the caries model group (*p* < 0.001). However, once CHX was stopped, the number of *S. mutans* continued rising, from 4.7 log (CFU/mL) (second sampling) to 5.3 log (CFU/mL) (fourth sampling), which was close to the number observed in the caries model group (*p* > 0.05), while the population of *S. mutans* in the oral cavity (P2) remained at a low level (4.3 log (CFU/mL), *p* < 0.001) until the end of the experiment after CCFM8724 administration was stopped.

A similar phenomenon was observed on the change of *C. albicans* (Figure 2(b)). The effect of CHX treatment (T1) on *C. albicans* tended to decrease with time. Moreover, CHX cannot prevent the colonization and growth of *C. albicans* (P1, *p* > 0.05), while CCFM8724 intervention exhibited a significant inhibitory effect on *C. albicans* (*p* < 0.001) during the experiment. Interestingly, the CCFM8724 prevention group (P2) did not immediately affect *C. albicans* after the colonization of *Lactobacillus* but reduced the number of *C. albicans* in the third (*p* < 0.05) and fourth (*p* < 0.001) sampling.

According to the counts of *L. plantarum* (Figure 2(c)), cariogenic bacterial and fungi did not affect the colonization of *Lactobacillus* (P2 and T2, *p* > 0.05), which ranged from 5.5 to 5.8 log (CFU/mL) in both two groups. The population of *L*. *plantarum* in the CCFM8724 prevention group (P2) experienced significant fluctuations in the second, third, and fourth sampling, from 4.7 (CFU/mL) to 3.7 log (CFU/mL), and back to 4.5 log (CFU/mL) (*p* < 0.05), while that in the CCFM8724 treatment group decreased from 5.8 log (CFU/mL) (second sampling) to 4.2 log (CFU/mL) (third sampling) and then stabilized to 4.2 log (CFU/mL) (fourth sampling).

### 3.2. Microflora Analysis

**Figure 3:**
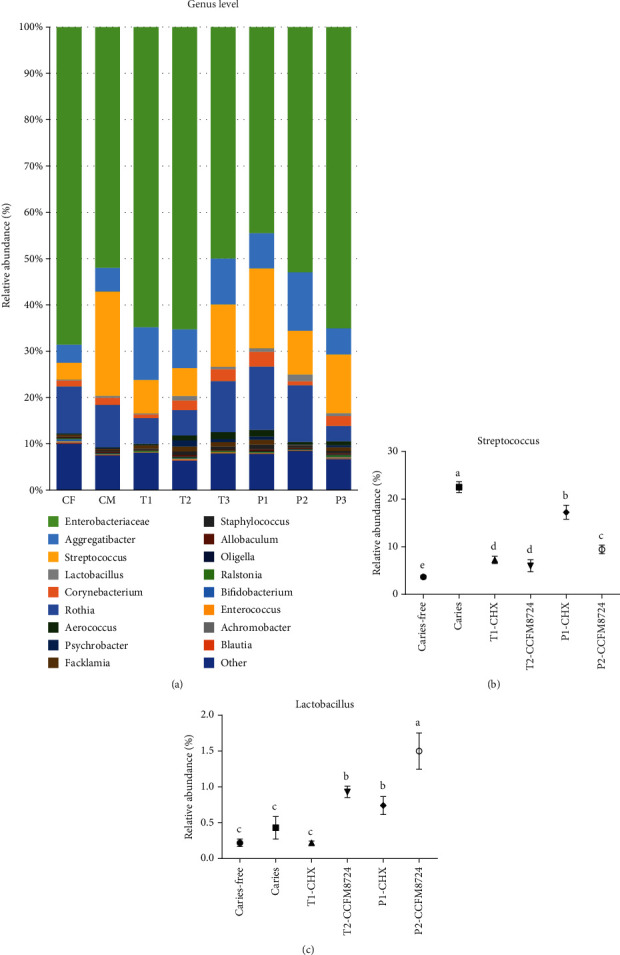
Changes in the level of genus in the oral cavity of rats in different groups. (a) Relative abundance of main genus > 0.1% in different groups. (b) Changes of the abundance of *Streptococcus* genus in different groups. Groups with dissimilar letters differ, *p* < 0.05. (c) Changes of the abundance of *Lactobacillus* genus in different groups. Groups with dissimilar letters differ, *p* < 0.05.

Since the counts of *S. mutans*, *C. albicans*, and *Lactobacillus* shifted in different groups during the experiment, we also revealed the alterations of bacterial abundance and proportion among different groups by Illumina 16S rDNA gene sequencing. Compared with the caries-free group, the application of 0.02% CHX or CCFM8724 revealed major changes in relative abundance in the oral microbiome of rats (Figure 3(a)). The notable difference in *Streptococcus* was found between the caries-free group (CF, 3.65%) and the caries model group (C, 22.5%) (*p* < 0.05). Treatment with CCFM8724 significantly decreased the abundance of *Streptococcus* when compared with the caries model group (*p* < 0.05), although there was no difference observed between the T1 (7.24%) and T2 (6.01%) groups (Figure 3(b)). The same degree of decrease was obtained in the two prevention groups (P1, 17.24%, P2, 9.43%, *p* < 0.05). Relative abundance of *Lactobacillus* remained lowest in the caries-free group (CF, 0.22%), caries model group (C, 0.43%), and CHX treatment group (T1, 0.22%). No significant difference was observed among these three groups. Treatment with CCFM8724 can significantly increase the relative abundance of *Lactobacillus*, as shown in Figure 3(b), while the *Lactobacillus* abundance in the CCFM8724 prevention group (P2) was more than 1%, which is the highest in all groups (1.5%, *p* < 0.05).

### 3.3. Micro-CT Analysis

**Figure 4:**
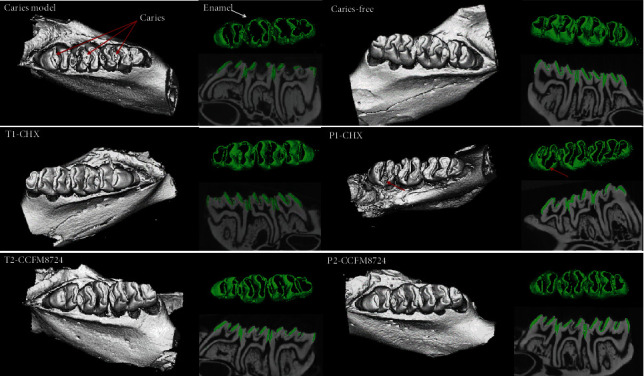
3D micro-CT image of mandibular molars, separated enamel (green), and corresponding 2D scale sagittal slice of the same molar (enamel is green) in each group. Red arrows: caries lesion site.

To enhance the visibility of caries site on rat molars, 3D reconstructions of mandible molars were performed by micro-CT. Enamel and dentin were stripped according to the density threshold (Hounsfield units). Meanwhile, the corresponding sagittal slice of the same molar was taken for comparative observation (Figure 4). Compared with the sagittal slice image between the caries model group and caries-free group, it is obvious that the enamel (green) of sulcal or adjacent areas were not continuous if caries occurred.

**Figure 5:**
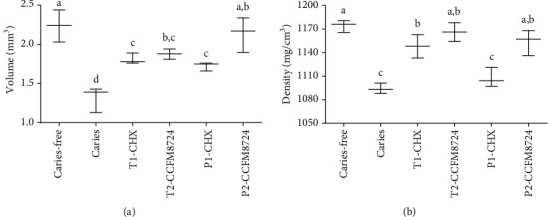
(a) Volume of enamel of mandibular molars. (b) MD of enamel of mandibular molars. Groups with dissimilar letters differ, *p* < 0.05.

To evaluate the results of micro-CT, the mean enamel volume (Figure 5(a)) and density (Figure 5(b)) of molars were analyzed in each group. Morphometric volume analysis revealed that the enamel volume in the CCFM8724 prevention group (P2) was closest to that of the caries-free group, followed by the CCFM8724 intervention group (T2). There was no significant difference in enamel volume between the CHX prevention group (P1) and intervention group (T1), but the enamel volume in both two groups was significantly larger than that of the caries model group (*p* < 0.05). The enamel density showed a similar result to those of enamel volume, but there was no difference in enamel density between the CHX prophylaxis group (P1) and dental caries model group (*p* > 0.05).

### 3.4. Caries Scoring

Under stereoscopic microscopy, various caries lesions were observed in all of the dyed molars except the caries-free group. The caries scores (Table 1) indicated that the lesion level E (enamel caries) of smooth surface or sulcal surface was considerably decreased in all the four groups, except that the CHX prevention group did not exhibit a significant effect on sulcal caries. According to the scores of different severity, the CCFM8724 prevention group showed the best effect, in which smooth surface and fissures did not appear with extensive caries (Dm, 3/4 of the dentin affected). And the total score (E) of this group was the lowest. The order of anticaries effect is as follows: the *Lactobacillus* treatment group, CHX treatment group, and lastly, CHX prevention group.

## 4. Discussion

In view of the increasing incidence and prevalence of dental cavity and its detrimental effects on oral health, novel strategies are required for its prevention and control. In recent years, the application of probiotics to prevent dental caries has become more and more common. Krzyściak et al. proved that *L. salivarius* HM6 could inhibit *S. mutans* and *C. albicans* dual biofilm formation and reduce the pathogenic species in *vitro* [29]. Although lactobacilli themselves could produce organic acid, it can be concluded that the overall effect of lactobacilli on caries prevention seems favorable when probiotics candidates are carefully selected [30]. And other studies have shown that *Lactobacillus* could attenuate the growth of *S. mutans* [31] or *C. albicans* [32] in human mouth. However, little is known whether *Lactobacillus* could exhibit effect on the mutually reinforcing alliances of *S. mutans* and *C. albicans* that coexist in the mouth.

In our study, CCFM8724 showed a considerable inhibitory effect on *S. mutans* and *C. albicans* during the intervention which lasted less than 2 months, three times a week (Figures 2(a), 2(b), and 3(b)). The prevention group in which CCFM8724 colonized firstly succeeded in controlling the proliferation of *S. mutans* but did not affect the colonization of *C. albicans* at first (Figures 2(a) and 2(b)). Although *C. albicans* decreased significantly and stabilized at a certain level in the later stage, this may be related to the protective effect of *S. mutans* on *C. albicans* [33, 34]. The colonization of *Lactobacillus* is also the key to its function. From Figure 2(c), it can be seen that the colonization of CCFM8724 was a fluctuating process, which may be the result of competitive adherence with pathogenic bacteria in the mouth [35]. Meanwhile, *Lactobacillus* could maintain a healthy oral environment by producing antimicrobial substances including organic acids, hydrogen peroxide, bacteriolytic enzymes, bacteriocins, and biosurfactants to inhibit the growth of pathogenic bacteria [36, 37]. However, the oral cavity is a nutrient fluctuating environment, whether CCFM8724 could maintain the balance of the oral environment for a longer time should be investigated further.

The oral microbiome analysis of the rats in different tested groups was a novel aspect to supplementarily certificate the CFU counting results. The genus level composition of all groups seemed no notable differences, and the major genus detected throughout the study were *Enterobacteriaceae*, *Aggregatibacter*, *Streptococcus*, and *Lactobacillus* as displayed in Figure 3(a). Meanwhile, the relative abundance of *S. mutans* in the caries model (22.5%) is in agreement to a recent report by Garcia et al. [38], in which the caries model was established by *S. mutans* alone. The diversity of oral microbiota in the caries model seemed to decrease, which may be attributed to the colonization of *Candida*. Haukioja et al. also found that increased *Candida* was related to reduced diversity of salivary microbiota [39]. Furthermore, the relative abundance of the *Lactobacillus* and *Streptococcus* in T2 and P2 (Figure 3(b)) groups was negatively correlated, which was consistent with the counting results in Figure 2. Intriguingly, *Aggregatibacter actinomycetemcomitans* has been reported to produce a signaling molecule called autoinducer-2 (AI-2) which could regulate the *C. albicans* biofilm formation [40]. Combined with the relative abundance changes of *Aggregatibacter* in the model group and other test groups, there may be a negative correlation between *Aggregatibacter* and *C. albicans*. More explorations should be taken to demonstrate this in further study. After all, *C. albicans* can affect the composition of bacteria in the oral cavity and its status in the oral ecosystem is no longer neglectable [41]. However, the microbiome of oral fungi in caries model needs to be further explored by gene sequencing.

Microtomography (micro-CT) is a modified version of medical computed tomography, which can capture images from multiple angles and produce nondestructive visualization of dental structures in three dimensions [42]. As a tool to provide high-resolution images as well as both qualitative and quantitative analyses of the tooth, microtomography is the gold standard for caries detection and evaluation in vitro, which gained increasing popularity in use for dental research [43]. Hence, mciro-CT has been recommended as a reliable method to evaluate the volume and MD of dental hard tissue, especially to focus on the density of the hard tissues [42, 44]. It is well known that caries occur with demineralization of hard tissues, and enamel is the initial site of dental caries. Therefore, the enamel which separated from the dentin is often used to analyze the caries severity in most studies [45–47]. In this study, the enamel volume and MD of one side of the mandible were calculated. The larger the volume of the residual enamel indicated the stronger the anticaries effect of the tested group. And the higher MD value proved the less loss of minerals or the stronger remineralization effect. And the result showed that the enamel caries severity increased in the order of P2, T2, T1, and P1, which provided evidence of the notable effect of *Lactobacillus*, while little effect of CHX prevention.

Despite these considerable advantages of micro-CT, the high cost and long scanning time are the disadvantages. Therefore, the Keyes scoring method is still widely used for analyzing the primary caries in animal models along with the supplementary testimony of micro-CT. Klinke et al. [22] revealed that the combination of *S. mutans* and *C. albicans* could lead to more serious pit and fissure caries than smooth caries, which is similar to the results of the model group (Table 1). No significant difference of smooth surface caries was observed in caries scores or images among four tested groups. What really widened the score gap was the relief of pit and fissure caries. Except that the CHX intervention group had no significant effect on occlusal caries, other groups showed a notable effect on smooth and fissure caries. The lowest total score (level E) was the CCFM8724 prevention group, which indicated that *Lactobacillus* exhibited better anticaries effect when colonized firstly in the oral cavity than later treatment. Probiotics can be promising candidates for novel anticariogenic substances owning to their essential ability of oral colonization and competition with oral pathogens for adhesion sites [35, 48].

In this study, both CCFM8724 treatment and prevention significantly attenuated the growth of *S. mutans* and *C. albicans* in the rat oral cavity, while CCFM8724 showed potential ability to colonize the oral cavity which is also the key to its function. In addition, the significant difference of caries lesions between the model group and lactobacillus-associated groups were verified via micro-CT and Keyes scoring method, demonstrating the anticaries properties of CCFM8724 *in vivo*. Further confirmation by clinical studies, would confirm the oral probiotic nature of CCFM8724 in alleviating dually infected caries.

## Figures and Tables

**Table 1:** Effect of different groups on caries development (incidence and severity) in rats.

Group	Smooth surface			Sulcal surface		
	Total	Severity		Total	Severity	
	E	Ds	Dm	E	Ds	Dm
Caries model	13.2 ± 5.2	8.3 ± 0.8	5.7 ± 0.5	23.7 ± 5.2	18.8 ± 4.1	9.3 ± 2.4
T1-CHX	8.6 ± 2.6^∗^	5.0 ± 1.1^∗^	2.1 ± 0.6^∗^	21.3 ± 6.8^∗^	12.9 ± 3.5^∗^	7.1 ± 1.4
T2-CCFM8724	6.6 ± 3.8^∗^	4.7 ± 1.1^∗^	2.0 ± 1.4^∗^	19.6 ± 4.0^∗^	6.6 ± 2.7^∗^	2.4 ± 0.8^∗^
P1-CHX	7.9 ± 5.6^∗^	6.5 ± 2.8	4.5 ± 2.1	21.8 ± 6.2	16.3 ± 4.9	6.9 ± 1.9
P2-CCFM8724	6.5 ± 2.4^∗^	1.9 ± 0.1^∗^	—	15.4 ± 7.3^∗^	3.1 ± 3.0^∗^	—

Data are expressed as mean ± standard error of the mean (*n* = 8). E: enamel caries; Ds: dentin exposed; Dm: 3/4 of the dentin affected. ^∗^*p* < 0.05 when compared with the caries model group.

## Data Availability

Data used to support the findings of this study are available from the corresponding author upon request.
